# Native Aorta Non-Touch Total Arch Hybrid Repair for Residual Aortic Dissection after Hemiarch Replacement

**DOI:** 10.3400/avd.cr.26-00112

**Published:** 2026-11-10

**Authors:** Kazuma Handa, Tomohiko Sakamoto, Toshihiro Ohata, Teruya Nakamura, Toru Kuratani

**Affiliations:** 1 Department of Cardiovascular Surgery, Sakurabashi Watanabe Advanced Healthcare Hospital, Osaka, Osaka, Japan; 2 Department of Cardiovascular Surgery, The University of Osaka Graduate School of Medicine, Suita, Osaka, Japan

**Keywords:** redo total arch repair, hybrid surgery, aortic dissection

## Abstract

Reintervention for residual aortic arch dissection after hemiarch replacement for acute type A aortic dissection remains technically challenging. A 70-year-old man presented with progressive dissecting arch aneurysmal dilatation 6 years after hemiarch replacement. Native aorta non-touch total arch hybrid repair was performed using the previously implanted hemiarch graft as the inflow source for total supra-aortic debranching and as the site for short elephant trunk placement. Staged thoracic endovascular aortic repair was performed the following day. Postoperative computed tomography showed no endoleak, and the patient remained free from aortic events at 5-year follow-up.

## Introduction

Hemiarch replacement is the standard surgical procedure for acute type A aortic dissection; however, residual arch dissection may progressively enlarge during follow-up and require reintervention.^1,2)^ Reoperation is technically demanding because severe adhesions increase the risk of bleeding and perioperative mortality,^3–5)^ and extensive aneurysmal degeneration of the residual aorta often complicates the identification of a suitable distal anastomotic site.^6)^ A single-stage arch repair using a frozen elephant trunk inserted through a previously implanted graft has been reported.^7)^ We report a patient who underwent hybrid arch repair combining total supra-aortic debranching and short elephant trunk (sET) placement, with the debranching graft anastomosed to the previously implanted hemiarch graft as the inflow source and the sET inserted into the same hemiarch graft, thereby avoiding extensive adhesiolysis and native aortic manipulation. This staged approach allows controlled and accurate deployment of the stent graft at the intended distal landing site under fluoroscopic and angiographic guidance, which may help reduce the risk of distal stent graft-induced new entry, while allowing distal aortic repair with consideration for preservation of spinal cord perfusion.

## Case Report

A 70-year-old man had undergone hemiarch replacement (J-graft 26 mm; Japan Lifeline, Tokyo, Japan) for acute type A aortic dissection with a patent false lumen 6 years earlier. Follow-up computed tomography (CT) showed enlargement of a dissecting aortic arch aneurysm to 55 mm and dilation of the brachiocephalic artery to 35 mm, indicating the need for surgical intervention (**Fig. 1**). Because severe adhesions were anticipated and no suitable distal anastomotic site was available, and the distal arch and descending aorta exceeded 45 mm in diameter, a native aorta non-touch total arch hybrid repair was planned. A redo median sternotomy was performed, and dissection was limited to the previously implanted hemiarch graft and the superior vena cava. Cardiopulmonary bypass (CPB) was established with venous drainage from the femoral vein and superior vena cava, and prosthetic grafts were anastomosed to both axillary arteries for arterial perfusion. After systemic cooling to 20°C, deep hypothermic circulatory arrest was instituted, without selective antegrade cerebral perfusion. A cervical debranching composite graft (J-graft debranch graft 11–9–9 for bilateral carotid arteries and the left subclavian artery, composed with an additional J-graft 9 mm×2 for the right subclavian artery and for arterial perfusion of CPB) was anastomosed end-to-side to the proximal portion of the hemiarch graft (**Fig. 2A**). The distal hemiarch graft was then incised semicircumferentially, and a sET (J-graft 26 mm–4 cm) was inserted into the graft and secured with sutures; the graft incision was then closed (**Fig. 2A**). The brachiocephalic artery origin was oversewn with felt reinforcement, and all supra-aortic branches were reconstructed (**Fig. 2B**). The operative time was 7 h 50 min, CPB time was 173 min, circulatory arrest time was 43 min, and intraoperative blood loss was 2700 mL. On postoperative day 1, thoracic endovascular aortic repair (TEVAR) was performed from the sET to the descending aorta using a conformable GORE TAG (CTAG; W. L. Gore & Associates, Flagstaff, AZ, USA) 37 mm–20 cm, completing the arch repair (**Fig. 2B**). To ensure coverage of the sET suture line, the stent graft was extended beyond the sET into the existing hemiarch graft. Because the 26-mm J-graft measured approximately 30 mm in inner diameter on preoperative CT, a 37-mm CTAG was selected. The stent graft was carefully deployed under fluoroscopic guidance while monitoring the sET configuration, with no eversion or inversion of the sET. The patient was extubated in the operating room. Postoperative CT showed no evidence of endoleak (**Fig. 3**). No major perioperative complications occurred, including stroke, spinal cord ischemia, or recurrent laryngeal nerve palsy. The patient was discharged home ambulatory 2 weeks after surgery. He remains well at 5-year follow-up. Annual CT follow-up was performed, and contrast-enhanced CT at 5 years confirmed patency of all branch grafts without endoleak, stent-graft migration, or other aortic events.

**Fig. 1:**
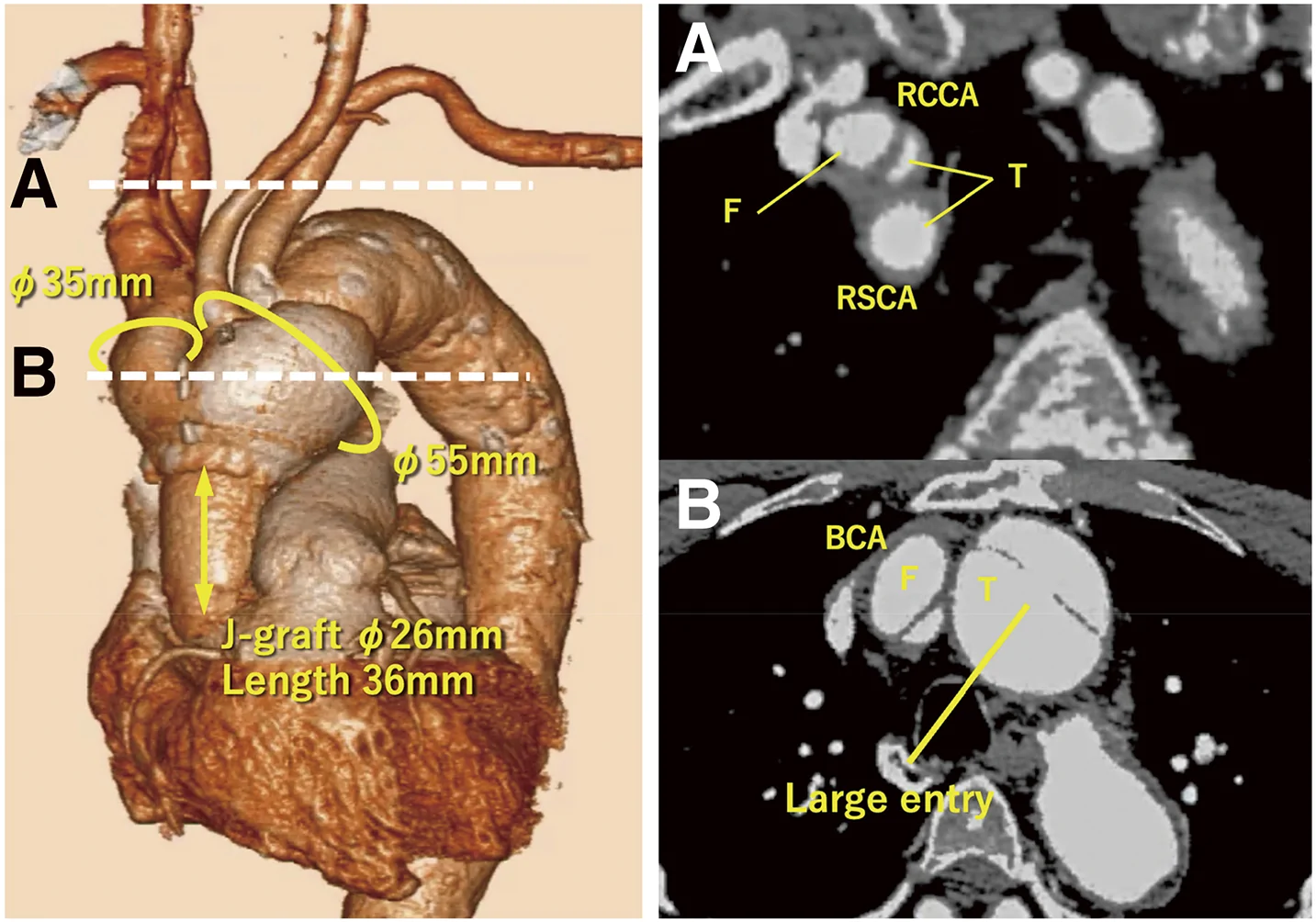
Preoperative computed tomography. BCA, brachiocephalic artery; F, false lumen; RCCA, right common carotid artery; RSCA, right subclavian artery; T, true lumen

**Fig. 2:**
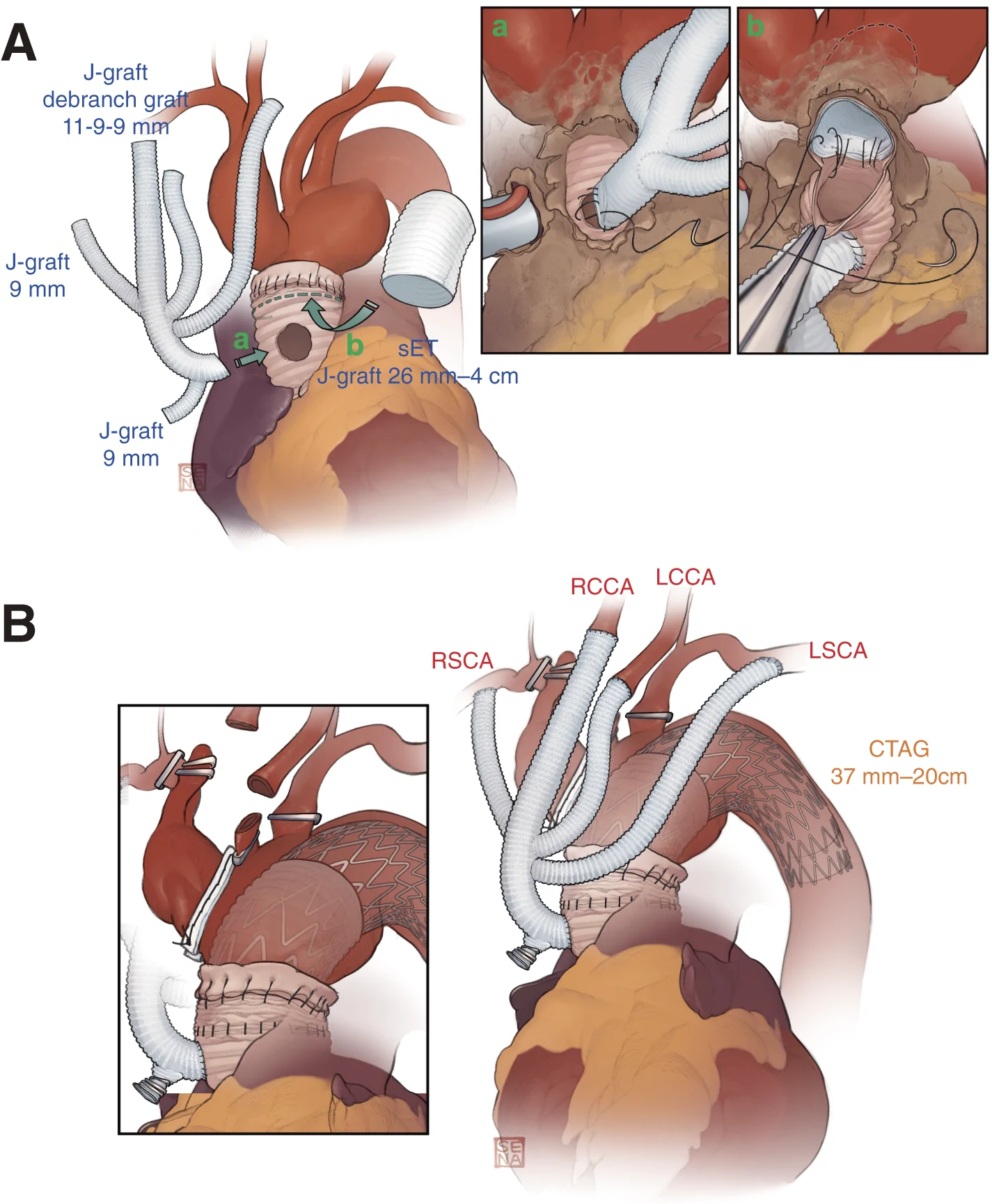
Schematic illustrations of the surgical procedure. (**A**) A cervical debranching composite graft (J-graft: Japan Lifeline, Tokyo, Japan, debranch graft 11–9–9 mm for RCCA, LCCA, and LSCA, composed with an additional J-graft 9 mm×2 for RSCA and for arterial perfusion of cardiopulmonary bypass) was anastomosed end-to-side to the proximal portion of the hemiarch graft, and a short elephant trunk (J-graft 26 mm–4 cm) was inserted into the existing hemiarch graft under circulatory arrest. (**B**) Thoracic endovascular aortic repair was performed from the short elephant trunk to the descending aorta using a conformable GORE TAG (CTAG; W. L. Gore & Associates, Flagstaff, AZ, USA) 37 mm–20 cm, completing the arch repair. LCCA, left common carotid artery; LSCA, left subclavian artery; RCCA, right common carotid artery; RSCA, right subclavian artery; sET, short elephant trunk

**Fig. 3:**
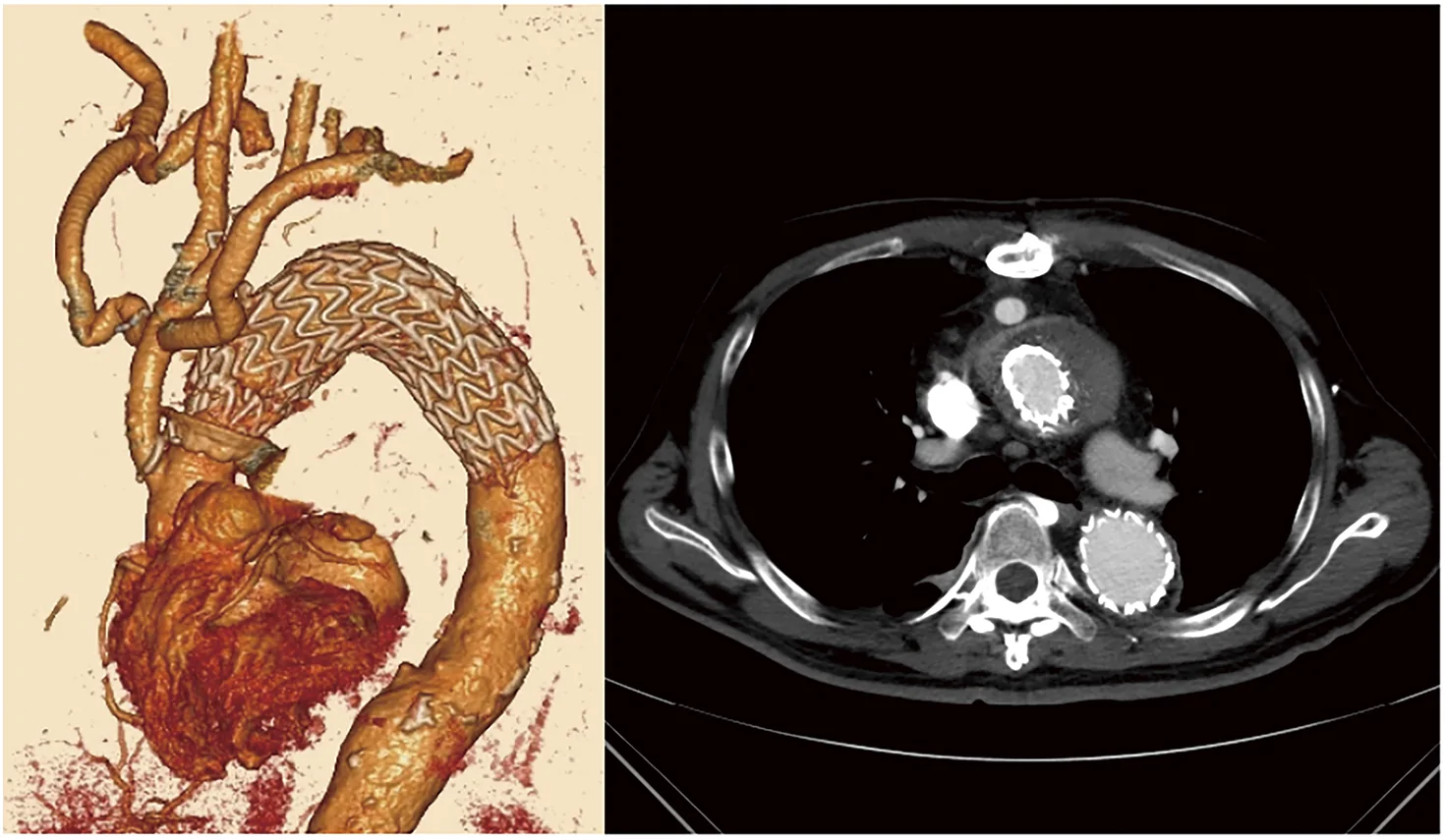
Postoperative computed tomography. Postoperative computed tomography showed no endoleak or stent graft-induced new entry and confirmed good patency of all reconstructed supra-aortic branch grafts.

Two additional patients treated with the same technique during the same period also had favorable 5-year outcomes and are described in the **Supplementary Cases**.

## Discussion

Hemiarch replacement is widely accepted as the standard surgical strategy for acute type A aortic dissection as it prioritizes life-saving intervention. However, in patients with a persistent false lumen, residual dissection of the aortic arch or descending aorta may progressively enlarge during follow-up and necessitate reintervention.^1,2)^ Redo total arch replacement (TAR) for residual dissecting arch aneurysm after hemiarch replacement remains technically demanding, as extensive adhesions and wide dissection are often required, resulting in substantial bleeding risk and increased operative morbidity.^3–5)^

Against this background, the present case demonstrates the feasibility of native aorta non-touch total arch hybrid repair for residual dissecting arch aneurysm after hemiarch replacement. The key concept is to limit dissection to the previously implanted hemiarch graft, thereby avoiding manipulation of the fragile, dissected native aorta and minimizing operative bleeding. Total supra-aortic debranching from the previously implanted hemiarch graft enables reconstruction of the supra-aortic branches, and insertion of a sET within the same graft provides a stable proximal landing zone for subsequent TEVAR.

The optimal extent of arch repair at the index operation remains controversial. A previous study suggested that TAR may reduce the risk of late distal aortic events.^8)^ However, contemporary multicenter data have associated TAR with higher perioperative mortality than more limited proximal repair, without reducing distal aortic reoperation.^9)^ In addition, a substantial proportion of patients undergoing hemiarch replacement never require subsequent arch reintervention.^1,2)^ Because the primary goal of surgery in the acute phase is to save the patient’s life, the rationale for selecting hemiarch replacement remains strong. Thus, the extent of initial repair should be tailored to the extent of disease, entry-tear location, supra-aortic branch involvement, and patient condition; partial arch replacement may represent an intermediate option.

Residual dissecting aneurysms after hemiarch replacement often enlarge from the arch to the descending aorta, frequently resulting in diffuse aortic dilatation. In such cases, identifying a distal segment suitable for safe anastomosis during conventional redo TAR can be particularly difficult. In the present case, the use of the previously implanted hemiarch graft as both the inflow source for debranching and the site for sET insertion helped circumvent this challenge.

Partial or frozen elephant trunk techniques enable single-stage treatment of the arch and proximal descending aorta, and Inoue reported single-stage open arch repair using a frozen elephant trunk inserted through a previously implanted graft.^7)^ However, frozen elephant trunk repair carries a risk of distal stent graft-induced new entry,^10)^ while extensive descending aortic coverage requires careful consideration of spinal cord ischemia. In our approach, a non-stented sET serves as the proximal landing zone for staged TEVAR, allowing distal aortic repair after postoperative bleeding and hemodynamics have stabilized. TEVAR also permits controlled and accurate deployment at the intended straight segment of the descending aorta under fluoroscopic and angiographic guidance, which may help optimize the distal landing zone and reduce the risk of distal stent graft-induced new entry. Compared with conventional redo TAR, this approach avoids extensive adhesiolysis, minimizes manipulation of the native dissected arch, including the region around the recurrent laryngeal nerve, and eliminates the need for a new anastomosis to the fragile native distal arch. Conversely, because the diseased native aorta is not resected, the procedure is less radical and carries potential long-term risks of proximal leakage, endoleak through intercostal or other collateral arteries, aortic reintervention, and stenosis or occlusion of the supra-aortic branch grafts. Accordingly, this technique should not be regarded as a general alternative to conventional TAR but as an option for selected patients with residual dissecting arch aneurysm after hemiarch replacement in whom extensive redo adhesiolysis or distal arch anastomosis is considered particularly high risk. Long-term CT surveillance for endoleak, residual aortic disease, and supra-aortic branch graft patency remains essential.

## Conclusion

Native aorta non-touch total arch hybrid repair utilizing the previously implanted hemiarch graft may be a feasible treatment option for selected patients with residual dissecting arch aneurysm after hemiarch replacement in whom extensive redo adhesiolysis or native distal arch anastomosis is considered high risk. Because the diseased native aorta remains in situ, long-term CT surveillance for endoleak, progression of residual aortic disease, and supra-aortic branch graft patency is essential.

## Supplementary Materials

Supplementary Cases Supplementary Materials include detailed descriptions of 2 additional patients treated with the same technique, together with preoperative and postoperative computed tomography images (**Figs. S1** and **S2**).

Fig. S1 Preoperative (**A**) and postoperative (**B**) computed tomography images of Case 2.

Fig. S2 Preoperative (**A**) and postoperative (**B**) computed tomography images of Case 3.

## Declarations

### Data availability

The authors declare that all data in this article are available within the article.
