# Supplementary material for: Native Aorta Non-Touch Total Arch Hybrid Repair for Residual Aortic Dissection after Hemiarch Replacement
Source: Ann Vasc Dis. 2026 Nov 10;19(1):26-00112. doi: 10.3400/avd.cr.26-00112 (PMC13751902; doi:10.3400/avd.cr.26-00112)
Supplement: Supplementary file 1 [file avd-19-1-26-00112-s001.pdf]

Supplementary Cases

Case 2: A 54-year-old woman had undergone hemiarch replacement (J-graft, 26 mm) for acute type A aortic dissection with a patent false lumen 7 years earlier. Follow-up CT demonstrated enlargement of a residual dissecting aortic arch aneurysm to 55 mm (**Fig. S1A**), warranting surgical intervention. Dissection was limited to the previously implanted hemiarch graft and the region around the superior vena cava. After establishment of cardiopulmonary bypass (CPB) and deep hypothermic circulatory arrest, a cervical debranching composite graft (J-graft debranch graft 11–9–9 mm with an additional 9-mm J-graft for arterial perfusion during CPB) was anastomosed end-to-side to the proximal hemiarch graft, and a 4-cm short elephant trunk (sET; J-graft, 24 mm) was inserted into the distal hemiarch graft and secured with sutures. All supra-aortic branches were then reconstructed. On postoperative day 1, thoracic endovascular aortic repair (TEVAR) was performed from the sET to the descending aorta using CTAG devices (28 mm × 20 cm and 34 mm × 10 cm). Postoperative CT showed no evidence of endoleak (**Fig. S1B**). The postoperative course was uneventful, and the patient was discharged home ambulatory 2 weeks after surgery. She remained well at 5-year follow-up.

Case 3: A 63-year-old man had undergone hemiarch replacement (J-graft, 26 mm) for acute type A aortic dissection with a patent false lumen 13 years earlier. Follow-up CT demonstrated enlargement of a residual dissecting aortic arch aneurysm to 55 mm (**Fig. S2A**), warranting surgical intervention. After establishment of CPB and deep hypothermic circulatory arrest, a cervical debranching composite graft (J-graft debranch graft 11–9–9 mm with an additional 9-mm J-graft for arterial perfusion during CPB) was anastomosed end-to-side to the proximal hemiarch graft, and a 4-cm sET (J-graft, 24 mm) was inserted into the distal hemiarch graft and secured with sutures. All supra-aortic branches were then reconstructed. On postoperative day 1, TEVAR was performed from the sET to the descending aorta using CTAG devices (31 mm × 15 cm and 28 mm × 10 cm). Postoperative CT showed no evidence of endoleak (**Fig. S2B**). The postoperative course was uneventful, and the patient was discharged home ambulatory 2 weeks after surgery. He remained well at 5-year follow-up.

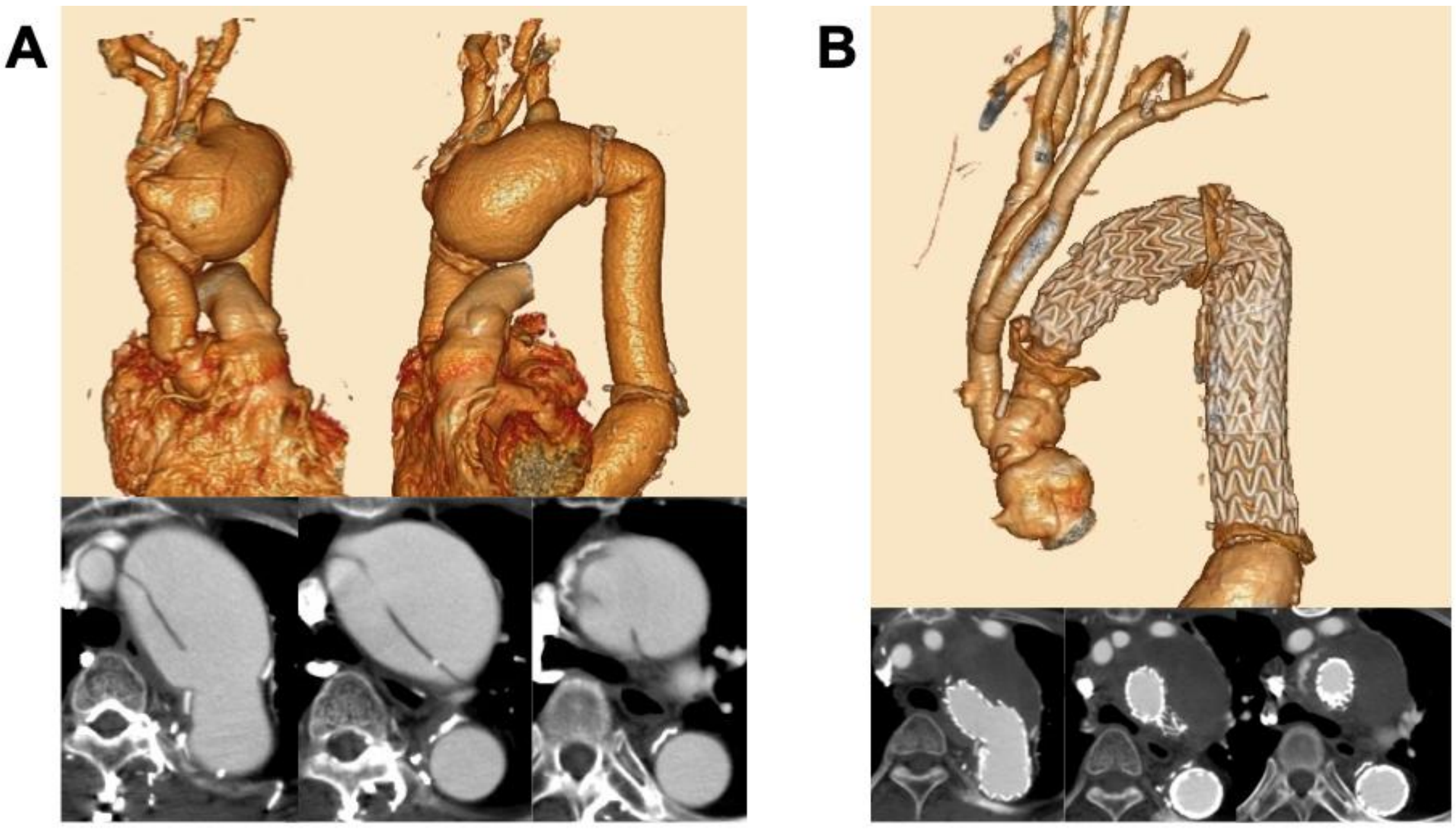

Fig. S1 Preoperative (A) and postoperative (B) computed tomography images of Case 2.

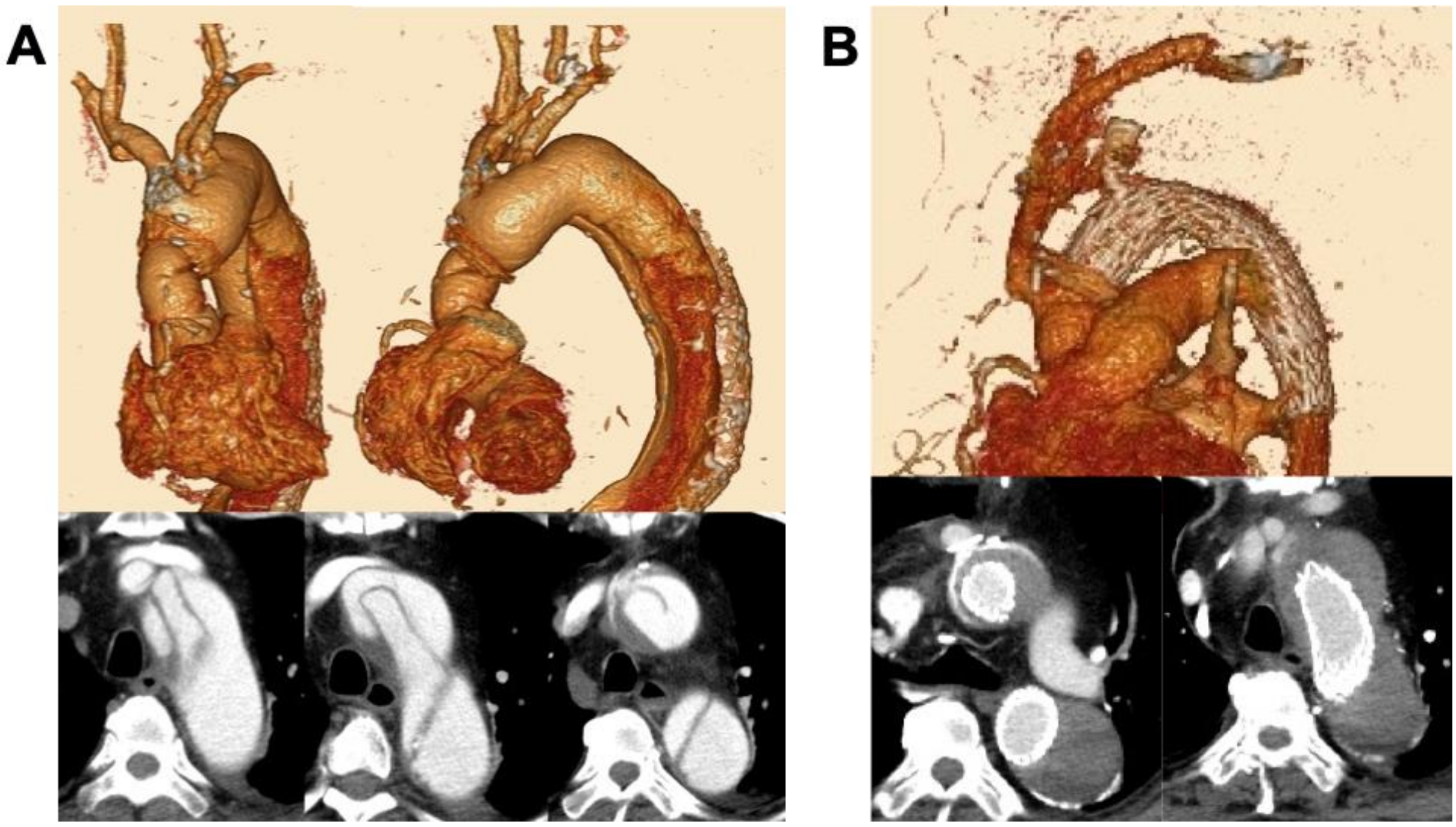

Fig. S2 Preoperative (A) and postoperative (B) computed tomography images of Case 3.
